# Lessons learned from new emerging infectious disease, Middle East Respiratory Syndrome coronavirus outbreak in Korea

**DOI:** 10.4178/epih/e2015051

**Published:** 2015-11-17

**Authors:** Joung Soon Kim

**Affiliations:** Graduate School of Public Health, Seoul National University, Seoul, Korea

## INTRODUCTION

Industrialization of transportation and human civilization has increased the frequency of travel and shortened the time of travel, resulting in many places around the globe within a day’s travel. Consequently, this human behavior has accelerated our risks for exposure to hitherto unknown pathogens.

In the human history of epidemic outbreaks, whenever hosts and pathogens made contacts for the first time, they have responded each other vigorously progressing to evolutionary adaptations through natural selection; the pathogens to the direction to lower virulence to survive without killing their hosts; coordinately the hosts adapt through individual and collective strengthening of immunity against the pathogens. Until such an evolutionary equilibrium is established, the clash usually triggers high-mortality pandemic episodes. Thus, any new infectious disease is hence a formidable threat to human lives.

Emergence of new pathogen, a mutant, and pathogen-host interaction may be different between various environments. In particular, changing environments that we are experiencing recently, may provide chances to rise new pathogenic mutants, which in turn provide opportunities of invading human hosts; for example people may pursue nature as a hobby by visiting jungles, deep seas, arctic regions, or in near future even other planets in the universe. In such cases, since the human body has not experienced these new pathogens in terms of immune system preparedness and response, the contact with new pathogens will result in massive occurrence of patients and deaths due to high infectivity, virulence and pathogenicity of the infectious agents. On the other hand, the infectious agent known through previously documented outbreaks, progressed to the certain degree of adaptation to human in other countries, such as the case with the recent Middle East Respiratory Syndrome (MERS) outbreak, should not pose any great problems, especially when even diagnostic tools have already been developed.

## REASONS FOR FAILING TO PROVIDE TIMELY MANAGEMENT OF MIDDLE EAST RESPIRATORY SYNDROME OUTBREAK

Middle East Respiratory Syndrome coronavirus (MERS-CoV) is known to have relatively low infectivity (by low incidence rate) and low virulence (by low fatality rates) among healthy individuals without underlying diseases. In Korea, however, early prevention and control failed resulting in an enormous social, economic and psychological impact nationwide. Why did this failure of public health occur in the most recent outbreak? Despite the risk involved in relying on only published papers and media without a direct field investigation, I have accepted an invitation to contribute written work on the subject as solicited by the Korean Society of Epidemiology, because of my long-standing experiences as an epidemiologist, frequently convened by the Ministry of Health and Welfare for field investigations, whenever the media raise issues about an unidentified infectious disease, hoping this work may be of help for future occurrence of epidemics that could pose even more serious problems.

For the purpose I would like to focus on necessary countermeasures and the reasons why the outbreak prevention, efficient outbreak control and management, failed. First, rapid recognition and confirmation of infected cases is essential for prevention. In recognizing new disease, physicians’ diagnostic ability is very important starting with only clinical features of the patient, which encompasses curiosity for the unknown disease, keeping diagnostic approaches open for a new infectious disease, and careful attention to patients’ complaints including a meticulous examination.

While it is boasted that Korea’s level of medical practice is comparable to those of any advanced countries, a current defect of the Korean medical system is that it does not currently reflect an appropriate remuneration of medical activities involving specialized competences, which leaves physicians pressed for time, unable to listen and examine their patient’s conditions thoroughly. If the physician who examines the first patient with a dangerous infectious disease fails to identify it, the system will falter. It is useless to build hospitals with specialized facilities in every region, spending a large amount of money, because efficient outbreak prevention and control is dependent upon the physician who recognized the very first patient and report the suspected case immediately to the authorities concerned. To enhance the general preparedness of each hospital against new infectious diseases, it is desirable to provide on-the-job training for the entire medical staff at least twice a year, as the first patient infected by a new infectious agent may visit any specialty of any medical facility. For on-the-job training, experts for training hospital staff may be invited from the infectious disease department of the Korea Centers for Disease Control and Prevention (KCDC) or the Korean Society of Epidemiology.

During the recent MERS outbreak, a great contribution was made by Samsung Medical Center in Seoul as they reported a suspected MERS case enabling confirmatory diagnosis, which was as much hard to diagnose as had missed by many other hospitals due to its non-specific clinical features.

Second, once a confirmatory diagnosis is made, immediate countermeasures are essential. In light of various social and political situations in Korea, the major reason for the prolonged period of MERS-CoV spread despite its relatively low infectivity appeared to be attributed to the lack of rapid and efficient cooperation between the hospitals where the first patient was under care, and the authorities responsible for infectious diseases, i.e. the KCDC, given that hesitation or delay of a few minutes at this juncture equates to hundreds of minutes in normal time. This hesitation has brought the disaster. If the KCDC had conducted an epidemiological investigation, and blocked traffic (e.g. immediate closure of the hospital accommodating the patient) immediately, this MERS epidemic could have been prevented; if a thorough quarantine of patients and contacted persons had been implemented immediately, the secondary and tertiary outbreaks in several locations would have been prevented.

Even though the Infectious Disease Prevention Act exists in Korea, local hospitals and residents are not very cooperative with epidemiologists even after they are informed these scientists have been sent by the Ministry of Health and Welfare. The low confidence among local residents towards health authorities (the Ministry of Health and Welfare, community health care), has shown a little improvement compared to the past, continues to interfere with the rapid implementation of appropriate outbreak prevention measures imposed with the support of the local governments. This should be improved by endowing stronger authority to field epidemiologists, Epidemic Intelligence Service (EIS) officers, and community health center staff in emergency cases such as an epidemic outbreak. In the recent MERS outbreaks, the failure seemed to be attributed to a lack of timely closure of the hospitals, a long hesitation, and delay in the complete isolation of the patients and contacted persons, a consequence of failed implementation of KCDC authority and responsibility. Additionally, the pride of the hospital authorities as clinical experts with knowledge, skills, strategies, and trouble-shooting capacities perceived as not inferior to epidemiologists and EIS experts also seems to have played an important role. In emergencies where public health is at stake, showing off pride and arrogance should be an absolute taboo. The spread of MERS-CoV kindled and fueled a huge wave of debates across the country, covered in the media and the National Assembly, as if it were a great national agenda. However, its prevention actually could have been only a matter of rapid and accurate implementation of the ABCs of disease prevention and control. If this fundamental principle can be adhered to, attacks of even the most formidable viruses would not pose issue at any time or place. In other words, if all citizens, villagers, and healthcare workers are alert to detect new diseases and report to the responsible authorities, which in turn take immediate actions necessary for prevention, there will be no more problems posed by epidemics of new infectious diseases.

## EPIDEMIOLOGICAL INVESTIGATION AND ANALYSIS SHOULD BE CONDUCTED AS FOLLOWS

(1) The clinical characteristics (e.g., initial signs and symptoms, clinical course, prognosis) and the natural history of the disease are identified to establish diagnostic criteria being followed by confirmatory test development; (2) the pathological characteristics (e.g., clinico-pathological tests findings, pathohistological findings, autopsy findings) are used for tracking the pathogens; (3) epidemiological characteristics (e.g., patient distribution patterns in a timeline, incidence and fatality rates by gender, age, region, environmental, and patient characteristics) are used for exploring the cause; (4) thoroughly exploration of unapparent infections and calculate infectivity (all infected persons/all exposed persons to the pathogens), pathogenicity (no. of pts/all infected persons), and virulence of the pathogens (no. of death from the infection/all infected persons) in order to estimate the outbreak impacts. The collected data should be analyzed and results should be integrated to track propagation paths in order to establish measures to block propagation, and establish outbreak prevention strategies. These measures and strategies are then applied to the populations at risk and already affected, and finally, their outbreak prevention efficacy is evaluated. Of course, it is a great challenge to implement all these tasks rapidly, accurately, and in a well-organized and coordinated manner, as they should be carried out through smooth cooperation among experts of numerous fields and institutions. Most of these tasks, however, are not always successfully achieved not because of a lack of knowledge and skills, but due to failure in executing the activities at the right place and time. Concisely, the main cause of failure is not owing to ignorance, but unfaithful attitude and flat negligence. Those who neglect their duties have their own reasons. However, any pretext is neither justifiable nor pardonable in situations where public health depends on the race against time in which seconds and minutes count. We have been too generous towards such paltry excuses so far in our disease prevention efforts. We should put an end to such generosity in order to end repeated disasters.

## CONCLUSION

We are at constant risk of exposure to new pathogens including the threats of bioweapons that may cause miserable disasters. In order to avoid the situation, all healthcare workers always open their eyes widely and focus their efforts on identifying undiagnosed, missed cases infected by pathogen suspicious of newly emerged. Immediate intuitive response of healthcare workers for every clinical episode suspicious of new disease entity is essential, and their task is completed only when immediate action is taken, case reporting, confirming diagnosis, participating prevention activities utilizing the best of their knowledge and skills under inter-agency, interdisciplinary, and inter-regional cooperation based on infectious disease management principles well known to them. Infectious disease management is like war, in which a moment of hesitation can determine victory or defeat. The recent MERS disaster should serve as a lesson that reminds us of the snowball effect of damages caused by failure to act in a timely manner while arguing over petty interests.

