# Supplementary material for: Lessons learned from new emerging infectious disease, Middle East Respiratory Syndrome coronavirus outbreak in Korea
Source: Epidemiol Health. 2015 Nov 17;37:e2015051. doi: 10.4178/epih/e2015051 (PMC4722224; doi:10.4178/epih/e2015051)
Supplement: Supplementary file 1 [file epih-37-e2015051-supplementary.pdf]

# 수입 전염병 메르스 유행발생의 교훈

김정순

서울대학교 보건대학원 명예교수

## 서론

교통 수단의 발달과 인류 문명의 발전은 지구촌을 일일 생활권으로 만들고 구석 구석 대부분의 지역을 쉽게 드나들 수 있게 되면서 우리가 전혀 경험하지 못했던 새로운 병원체에의 노출 기회도 헤아릴수 없이 많아 졌다. 인류의 감염병 발생사를 보면 어떤 새로운 병원체가 전혀 그 병원체에 접촉했던 경험이 없는 인구에 침입하면 병원체와 숙주인 인간집단이 한동안 맞 부디쳐서 병원체에게는 숙주를 죽이지 않고도 함께 살아갈 수 있을 만큼 병원성이 약화되는 방향으로의 진화를, 숙주는 병원체가 침입 못하도록 개인적, 집단적 면역력 증강방향으로의 변동으로 병원체와 숙주간의 평형이 이루어 질 때까지 치사율이 매우 높은 무서운 역질이 된다. 그래서 신종전염병은 무서운 존재이다.

이러한 병원체-숙주간의 상호 관계는 환경에 따라서도 변화한다. 특히 환경의 변화는 새로운 병원체 변이를 만들어 내기도 하고 인간집단에 유입시키는 계기가 되기도 한다. 예를 들면 문화적인 측면에서 사람들의 취미생활 영역의 확대로 정글 속 동물들 사이에서만 왕래하며 생존하던 병원체가 인간을 감염시키고 이것이 인간집단에 전파되는 경우 이다. 이런 경우에 인간에게는 대처방법이 미비하고 면역체계도 방어태세가 되어 있지 않아 감염되면 치명률이 높을 수 밖에 없다. 정글 뿐 아니라 북극에서, 혹은 우주 다른 별에서 올 수도 있다. 또 심해바닥에 잠겨 있던 병원체일 수도 있다. 금번 메르스처럼 이미 다른 나라에서 인간집단에 유행 경험이 있어 병원체가 이미 알려지고, 더구나 진단방법까지 개발된 병원체에 의한 유행은 크게 문제 될 것도 없다.

## 메르스 조기 방역에 성공 못한 이유와 그 효율적 관리방안

메르스는 병원체 특성상 감염력도 비교적 낮고 기저질환이 없는 건강한 사람에서는 치명률이 높지 않은 질병으로 알려져 있다. 그런데도 초기방역에 실패하여 사회적으로 큰 물의를 일으킨 이유는 무엇일까? 직접 현지조사도 못하고 발표된 논문과 언론 만 참고로 논란한다는 것이 다소 불안하지만 그 동안 소위 괴질이 발생했다고 언론에서 문제 제기가 될 때마다 보건복지부로 부터 동원명령을 받고 현지조사를 했던 경험에 비추어 무엇이 문제였는지 쉽게 추리 할 수 있기 때문에 미래에도 드물지 않게 발생할 더 어려운 사태에 참고가 되리라 믿고 원고를 청탁한 역학회의 제안을 받아들였다. 제한된 지면관계로 이러한 상황에서 어떻게 할 것인지, 그 원칙을 중심으로 방역실패의 원인/이유를 들여다 보고 이런 사건의 효율적 관리방안을 검토해 보고자 한다.

첫째 신속한 사건발생의 인지와 확인이다. 일선에서 진료하는 의사의 새로운, 또는 이전에 경험해 보지 못한 낯선 병을 인지하는 의사의 능력(새로운 것에 대한 호기심/새로운 전염병의 가능성을 염두에 둔 진료행위/항상 성의 있게 환자의 호소에 귀 기울이고 세밀히 관찰하는 진료습관 등등)이 중요하다. 우리나라 의료수준이 어느나라 못지 않게 높다고 자랑이 대단하지만 의사의 전문성을 고려한 의료행위에 대한 수당이 전혀 산정되지 않는 한국 의료제도의 결함 때문에 항상 시간에 쫓기는 의사들은 환자의 호소에 귀 기울이고 세밀히 관찰할 여유가 없다. 아무리 위험한 전염병이 한국에 들어와도 처음 환자를 진료한 의사가 이를 인지 못하고 놓친다면 아무리 고가의 전염병 전문병원을 지역마다 지어놓아도 소용이 없다. 진료의사가 새로운 병을 의심해서 당국에 신고하고 협조를 구해야만 효율적 방역사업이 시작되기 때문이다. 어떤 전염병이 병원 어느 전문과에 처음 올지 모르므로 일년에 적어도 두 번은 전 의료진을 대상으로 on the job training 을 하는 것이 바람직하다. 질병관리본부의 감염병센터나 한국역학회의 역학 전문가를 초청하면 좋을 것이다. 금번 서울 삼성병원에서 비특이적인 임상적 특성 때문에 새로운 질병이라고 인지하기  어려운 메르스 환자를 인지하고 신고하여 확진을 신속히 할 수 있게 한 것은 큰 공헌이다.

둘째 확진 뒤 신속한 조치이다.  감염력이 비교적 낮은 병원체인데도 만연된 이유는 한국의 여러 사회적 정치적 환경에 비추어 볼 때, ○1환자가 입원하고 있는 병원과 전염병관리의 책임을 지고 있는 관청, 즉 질병관리본부 체계와의 신속한 협조가 원활하지 못했던 점이다- 이 시점에서의 수 분간 주저/지연은 통상적 시간의 몇 백배에 해당하는 긴 순간이 되기 때문이다. 질병관리본부가 바로 역학조사를 수행하고 교통차단(환자가 있는 병원의 즉각적 폐쇄 등), 환자 및 접촉자의 격리수용을 철저히 수행 했더라면 2차, 3차 유행이 여기 저기서 발생하지 않았을 것이다. ○2우리나라에 엄연히 감염병예방법이 있는데도 현지에 가서 방역을 위해 보건복지부에서 파견된 요원이라고 설명해도 병의원이나 주민들은 적극적으로 응하지 않고 적대시 하는 경우가 많다. 전 보다는 많이 나아졌지만 아직도 보건복지부, 보건소에 대한 주민들의 신뢰감이 낮아 지방자치 정부의 행정부 협조를 통한 방역조치도 바라는 바 대로 신속히 수행되지 않는다. 역학조사관이나 방역요원, 보건소 직원들에게 전염병 유행 등의 비상시에는 좀 더 강력한 공권력이 부여되어 즉각 실행되어야 한다. 금번 메르스 유행때 병원의 폐쇄, 환자와 접촉자들의 철저한 격리가 되지 못한 이유도 질병관리본부의 권위와 책임이 제대로 인정되고 받아 들여지지 않고 장기간 머뭇거렸기 때문인 것으로 해석된다. 병원은 병원대로 임상의사의 입장에서 자신들의 지식, 기술, 전략, 문제해결 능력이 전염병관리 전문가들 만 못하지 않다는 자만심도 작용한 듯 보인다. 국민 건강이 걸려있는 긴박한 시기에 이러한 자만심과 오기는 금물이다. 언론에서 수십회에 걸친 토론회, 학회, 국회 등에서 무슨 대단한 사건인 것처럼 너도 나도 많은 논란들을 했는데 언제 어떤 더 무서운 역질이 들어와도 원칙만 신속하고 철저하게 시행하면 문제 될 것이 없다. 즉, 온 국민이, 온 의료인들이 항상 감시의 눈을 크게 뜨고 낯선, 새로운 질병을 잡아내고, 역학조사로 그 질병의 (1) 임상적 특성(초발증상 및 증후, 임상경과, 예후 등), 자연사를 파악하여 이를 기반으로 진단 기준을 설정한다. (2) 병리적 특성(임상검사 소견, 병리조직 검사결과, 부검 병리 소견)을 참조하여 원인 병원체

를 추리하는데 활용한다. (3) 역학적 특성(일시별 환자 분포 양상, 성별, 연령별, 지역별, 환경 특성별, 그리고 행태 특성별 발생률, 사망률, 치명률 등)을 근거로 원인의 탐색, 병원체의 감염력, 병원력, 그리고 독력을 산출하여 유행의 위험도를 추정한다. 한편 수집된 자료를 분석하고 결과를 종합하여, 전파경로를 추정하여 전파차단 방법을 최종 결정하여 방역대책을 수립, 현장에 활용하는 절차를 거쳐, 방역효과를 평가한다. 물론 이러한 업무는 여러 분야의 사람들과 기관의 원활한 협력으로 이루어져야하는 것이기 때문에 쉽지 않다. 많은 부분 우리가 몰라서 못하기 보다는 알면서도 적시 적소에 필요한 행위를 수행하지 않기 때문에 성취 못하는 경우가 대부분이다. 즉, 알면서도 안하기 때문이다. 그렇게되는 경우에는 그럴듯한 이유가 많다. 그러나 초와 분을 다투는 위급한 처지에 변명은 있을 수 없다. 우리는 그 동안 이러한 너절한 변명에 너무 관대해 왔다. 그래서 이런 불행이 반복 발생하는 것이다.

결어

어느때 어떤 새로운 전염성 병원체가 침입할지 모르는 상황에서 모든 보건 의료계 전문가들은 눈을 크게 뜨고 감시를 게을리 하지 말아야 하며 사태가 발생하면 즉각 우리가 알고 있는 관리원칙에 입각하여 기관간, 전문분야간, 지역간 원활한 협동하에 지식과 기술을 발휘하여 과감히 조처해야한다. 전염병관리는 전쟁과 같아서 순간의 머뭇거림이 승패를 결정하므로 이번 메르스의 경우처럼 작은 이해 때문에 머뭇거리다 실기하여 수십 배의 손실을 초래할 수도 있음을 명심해야 한다.
